# Comparison of Different Tyrosine Kinase Inhibitors for Treatment of Poor Performance Status Patients with EGFR-Mutated Lung Adenocarcinoma

**DOI:** 10.3390/cancers14030674

**Published:** 2022-01-28

**Authors:** Chiao-En Wu, Ching-Fu Chang, Chen-Yang Huang, Cheng-Ta Yang, Chih-Hsi Scott Kuo, Ping-Chih Hsu, John Wen-Cheng Chang

**Affiliations:** 1 Division of Hematology-Oncology, Department of Internal Medicine, Linkou Chang Gung Memorial Hospital, Chang Gung University College of Medicine, Taoyuan 333, Taiwan; mr0826@cgmh.org.tw (C.-F.C.); 9202070@cgmh.org.tw (C.-Y.H.); 2 Division of Thoracic Oncology, Department of Internal Medicine, Linkou Chang Gung Memorial Hospital, Chang Gung University College of Medicine, Taoyuan 333, Taiwan; yang1946@cgmh.org.tw (C.-T.Y.); r5245@cgmh.org.tw (C.-H.S.K.); 8902049@cgmh.org.tw (P.-C.H.)

**Keywords:** poor performance status, lung adenocarcinoma, afatinib, erlotinib, gefitinib

## Abstract

**Simple Summary:**

Epidermal growth factor receptor tyrosine kinase inhibitors (EGFR-TKIs) are standard treatments in patients with EGFR-mutated lung adenocarcinoma. However, the clinical data regarding EGFR-TKI efficacy in patients with poor performance status (PS ≥ 2) are limited. We reviewed the clinical outcomes and safety of EFGR-TKI use in patients with poor PS and identified the independent and favorable prognostic factors for progression-free survival and overall survival. We found that patients treated with 40 mg afatinib had better survival results, although only a non-significant trend toward superiority was observed in the multivariable analysis. Dose adjustment was an independent prognostic factor for PFS and OS. This study provided evidence of the use of EGFR-TKIs for patients with poor PS.

**Abstract:**

The aim of this retrospective study was to investigate the tolerability and survival outcomes of epidermal growth factor receptor tyrosine kinase inhibitors (EGFR-TKIs) treatment in patients with a performance status ≥ 2. The data for 517 patients treated with EGFR-TKIs between January 2011 and January 2018 at a regional hospital in northern Taiwan were analyzed. Clinical and pathological features were collected, and univariate as well as multivariable analyses were undertaken to identify potential prognostic factors. The overall objective response rate, median progression-free survival (PFS), and median overall survival (OS) were 56.3%, 11.4 months, and 15.3 months, respectively. The mutation status (exon 19 deletion), locally advanced disease, dose adjustment, and the lack of liver and pleural metastasis were independent and favorable prognostic factors for PFS. Age < 60 years, mutation status (exon 19 deletion), dose adjustment, and lack of lung, liver, and no pleural metastasis were independent and favorable prognostic factors for OS. GFR-TKIs demonstrated acceptable efficacy and safety in the current cohort. Dose adjustment was identified as an independent prognostic factor for both PFS and OS, regardless of which EGFR-TKIs were used. The current research provided novel evidence of the clinical prescription of frontline EGFR-TKIs for EGFR-mutated lung adenocarcinoma patients with a PS score ≥2.

## 1. Introduction

Advances in genetic research, drug development, and clinical trials have resulted in the recommendation that patients diagnosed with advanced non-small-cell lung cancer (NSCLC) who harbor activating mutations in the epidermal growth factor receptor (EGFR) gene be treated with tyrosine kinase inhibitors (TKIs) as the first-line treatment [1,2]. The currently recommended EGFR-TKIs include first-generation, such as gefitinib and erlotinib [3,4,5,6], and second-generation, such as dacomitinib and afatinib, which act as pan-human EGFR (HER) family inhibitors that irreversibly bind to EGFR [7,8,9,10]. The acquired T790M resistance mutation accounts for more than half of the resistance mechanisms identified in NSCLC patients undergoing treatment with first- and second-generation TKIs; therefore, a third-generation treatment, osimertinib, has been developed, which is effective against both EGFR-TKI sensitizing and resistance mutations (T790M) and demonstrated activity in patients who acquired T790M mutations following previous EGFR-TKIs treatment [11]. Although osimertinib demonstrated excellent survival outcomes contrasted with standard treatments (erlotinib or gefitinib) when used as a frontline treatment [12,13], first- and second-generation TKIs remain widely used in daily practice because sequential TKI treatment may benefit patients who develop T790M mutation, and this strategy is cost-effective [14,15].

Although second-generation TKIs showed better survival outcomes than first-generation TKIs, increased toxicities limited their clinical use in real-world experience [9,10]. In a large retrospective cohort study in Taiwan, patients treated with afatinib were younger and were less likely to have an Eastern Cooperative Oncology Group (ECOG)-performance status (PS) = 2 compared with those treated with gefitinib or erlotinib, the implication being that a physician’s TKI preference can be based on differing population groups. Before the era of TKIs, chemotherapy did not benefit patients with ECOG-PS > 2 due to a lack of evidence at the time, and best supportive care was suggested for such patients [16]. TKIs are generally well-tolerated, and the ECOG-PS score is no longer a predictive factor for determining TKI administration. However, patients with ECOG-PS > 2 are typically excluded from clinical trials and most retrospective studies [17,18,19,20,21]. Whether the second-generation TKI afatinib is a feasible treatment plan for ECOG-PS ≥ 2 NSCLC patients as opposed to first-generation gefitinib and erlotinib, when efficacy and safety are factored, remains unknown. Therefore, this study aimed to investigate the safety and efficacy of various EGFR-TKIs treatments in patients diagnosed with EGFR-mutated NSCLC and poor PS (PS ≥ 2).

## 2. Materials and Methods

### 2.1. Patients and Data Collection

**Figure 1:**
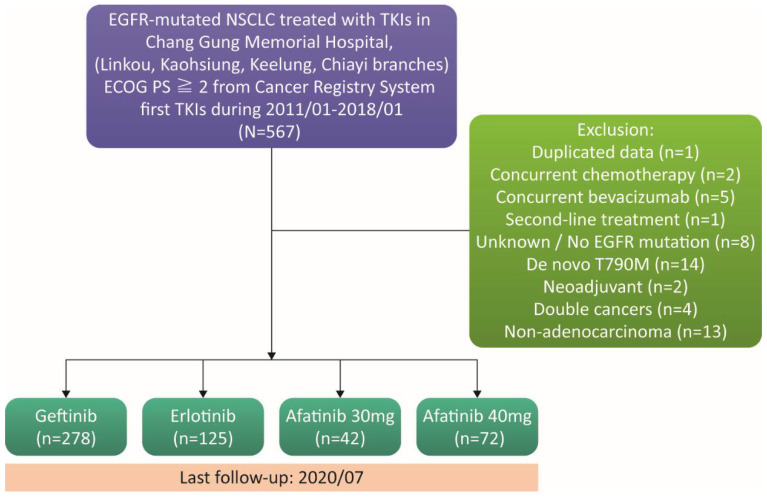
A flowchart showing patient enrollment and follow-up.

Patient data were obtained from the Cancer Registry System using the Chang Gung Research Database [22]. This study enrolled a total of 567 patients who were diagnosed with lung cancer; were assessed as ECOG-PS ≥ 2; harbored EGFR mutations; and were treated with EGFR-TKIs from January 2011 to January 2018. As this study aimed to examine patients treated with EGFR-TKI monotherapy for their first-line systemic treatment, those patients treated with concurrent chemotherapy (*n* = 2), concurrent bevacizumab (*n* = 5), second-line systemic treatment (*n* = 1), or neoadjuvant treatments (*n* = 2) were excluded. EGFR mutation status was retrospectively reviewed. Patients with a de novo T790M mutation (*n* = 14) and no or unknown EGFR mutation (*n* = 8) were excluded. Four patients with another active cancer and 13 patients with a non-adenocarcinoma history were excluded. Five hundred and seventeen lung adenocarcinoma patients treated with different EGFR-TKIs as first-line treatments were analyzed in this study, including 278, 125, and 114 patients treated with gefitinib, erlotinib, and afatinib, respectively. Gefitinib was prescribed at 250 mg daily and erlotinib was prescribed at 150 mg daily as a starting dose. The afatinib group was divided into two subgroups according to whether the starting dose was 30 mg (*n* = 42) or 40 mg (*n* = 72) daily. The patient selection process is summarized in Figure 1.

The clinical data of 517 patients who received first-line EGFR-TKIs were retrospectively reviewed. The clinicopathological features, including age; sex; smoking history; ECOG-PS score; tumor involvement; EGFR mutation, including exon 19 deletion, L858R, or uncommon mutation; dose adjustment (reduction/interruption); drug discontinuation; clinical response; adverse events (AEs) of EGFR-TKIs, and follow-up treatment were obtained. The final follow-up time point for this study was July 2020.

### 2.2. Treatment and Response Evaluation

The treatment of EGFR-TKIs ended with occurrence of disease progression or intolerable toxicity. The adjustments of prescription for EGFR-TKIs were made by physicians based on the tolerability of EGFR-TKI-related AEs. The tumor response was assessed by radiological studies, particularly by computed tomography. The Response Evaluation Criteria in Solid Tumors 1.1 (RECIST 1.1) were used to determine the clinical response, which was recorded as progressive disease (PD), stable disease (SD), partial response (PR), or complete response (CR). Tumor responses that were not assessed were recorded as “not assessed (NA)”. Progression-free survival (PFS) was the duration from EGFR-TKI treatment until the first radiological evidence of disease progression or the last dose of EGFR-TKI. Patients with no progression and no death during treatment were censored during the PFS analysis. Those patients who experienced radiological progression or death within one month after EGFR-TKI discontinuation and received no sequential treatment were counted as an event. OS was the duration from EGFR-TKI treatment until the last follow-up or death. The data of patients who did not expire were censored during OS analysis.

### 2.3. Statistical Analysis

Data regarding AEs were graded according to National Cancer Institute Common Terminology Criteria for Adverse Events, version 4.0. Dose adjustments (reductions or interruptions) and drug discontinuations or withdrawals due to AE occurrence were recorded.

### 2.4. Nomogram Creation and Statistical Software

The distribution of baseline characteristics, mutation types, metastatic sites, tumor responses, AEs, and the subsequent treatments received by study patients among the different TKI treatments were analyzed using the Kruskal–Wallis test for continuous variables and Fisher’s exact test for categorical variables. A series of univariate Cox proportional hazard models were conducted to initially screen for potential factors associated with PFS and OS. Those variables with significance values less than 0.15 in the univariate Cox analysis were further introduced into a multivariable Cox model [23]. Finally, pairwise comparisons among different TKI treatments for PFS and OS were stratified by several baseline characteristics, including age, sex, mutation type, stage, smoking, ECOG-PS, dose adjustments, drug discontinuations, and metastatic sites. A result was considered significant when *p* value < 0.05. SAS version 9.4 (SAS Institute, Cary, NC, USA) was used to perform all statistical analyses.

## 3. Results

### 3.1. Patient Characteristics

In this study, a total of 517 EGFR-mutated lung adenocarcinoma patients with ECOG-PS ≥ 2 who were treated with either gefitinib, erlotinib, or afatinib as first-line systemic treatments were included. A total of 278, 125, and 114 patients received gefitinib, erlotinib, and afatinib, respectively. The starting dose of afatinib for 42 patients was 30 mg daily, whereas 72 patients started at 40 mg daily. The median age of all included patients was 73.1 years (interquartile range [IQR]: 61.9 to 80.3 years), and 198 (38.3%) were men. The majority (*n* = 500, 96.7%) had Stage IV disease, based on the American Joint Committee on Cancer Staging System 7th Edition. EGFR mutations were identified, showing that 207 (40.2%) had an exon 19 deletion, 258 (50.1%) had an L858R mutation, and the remainder were classified as uncommon mutations (*n* = 50, 9.7%). No smoking history was reported for 398 patients (77.0%). A PS score of 2 was identified in 320 (61.9%) patients, and a PS score > 2 was identified in 197 (38.1%) patients. The bone was the most commonly identified metastatic site (55.1%), followed by the pleura (48.2%), brain (45.3%), and lung (43.5%). Significant differences in age, sex, dose adjustment, drug discontinuation, and brain metastasis were observed among the four treatment groups (*p* < 0.05). As expected, the patients receiving 40 mg afatinib were the youngest among all groups examined. The results showed that patients treated with 40 mg afatinib (median age of 65.2 years) were significantly younger than those who received gefitinib (median age of 73.1 years) and erlotinib (median age of 75.1 years). Men featured more in the erlotinib group when compared to the 30 mg afatinib group. Patients receiving 40 mg afatinib were more likely to require dose adjustment (50.0%) or drug discontinuation (15.3%) compared with those treated with gefitinib. No significant differences in other characteristics were observed among the treatment groups. All baseline characteristics for patients treated with the different EGFR-TKIs are summarized in Table 1 and Table S1.

**Figure 2:**
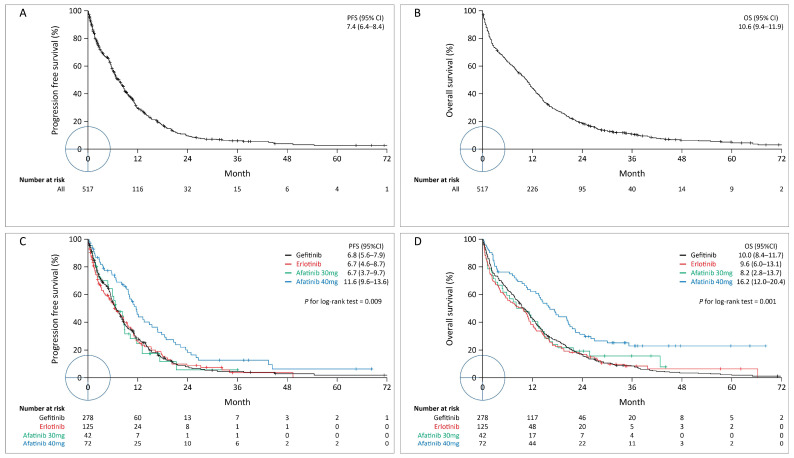
Kaplan–Meier survival curves indicating PFS (**A**,**C**) and OS (**B**,**D**) for all patients (**A**,**B**) and stratified according to the different EGFR-TKIs (**C**,**D**). EGFR-TKIs, epidermal growth factor receptor tyrosine kinase inhibitors; OS, overall survival; PFS, progression-free survival.

By the end of July 2020, the median follow-up time was 10.5 months. The median PFS (mPFS) and median OS (mOS) were 11.4 months (95% CI: 10.0–12.8 months) and 15.3 months (95% CI: 13.7–16.9 months), respectively (Figure 2A,B). The objective response rate (ORR) calculated as CR + PR was 56.3%, and the disease control rate (DCR) calculated as CR + PR + SD was 67.8%. The patients treated with 40 mg afatinib had favorable PFS and OS values compared with those treated with the other EGFR-TKIs (Figure 2C,D).

### 3.2. Prognostic Factors for PFS

Of the 517 patients, 411 (79.5%) experienced disease progression during a median follow-up of 5.8 months (IQR: 1.8–11.5 months; Figure 2A). Univariate analysis was performed using the univariate Cox proportional hazards model to detect prognostic factors for PFS in TKI-treated patients. Patients receiving an intial dose of 40 mg afatinib (vs. gefitinib, hazard ratio [HR]: 0.62, 95% CI: 0.47–0.81, *p* = 0.001), underwent dose adjustment (HR: 0.46, 95% CI: 0.36–0.59, *p* < 0.001), and experienced drug discontinuation (HR: 0.38, 95% CI: 0.16–0.87, *p* = 0.021) had favorable PFS. Patients who had L858R (vs. exon 19 deletion, HR:1.34, 95% CI: 1.09–1.64, *p* = 0.006) or uncommon mutations (vs. exon 19 deletion, HR:1.47, 95% CI: 0.99–2.17, *p* = 0.054), metastatic lung cancer (vs. locally advanced lung cancer, HR: 2.76, 95% CI: 1.48–5.15, *p* = 0.002), lung metastasis (vs. no lung metastasis, HR:1.29, 95% CI:1.06–1.56, *p* = 0.011), liver metastasis (vs. no liver metastasis, HR: 1.59, 95% CI:1.27–1.99, *p* ≤ 0.001), pleural metastasis (vs. no pleural metastasis, HR:1.32, 95% CI:1.08–1.60, *p* = 0.006), and other metastasis (HR:1.26, 95% CI:1.01–1.57, *p* = 0.039) had unfavorable PFS outcomes (Table 2).

**Figure 3:**
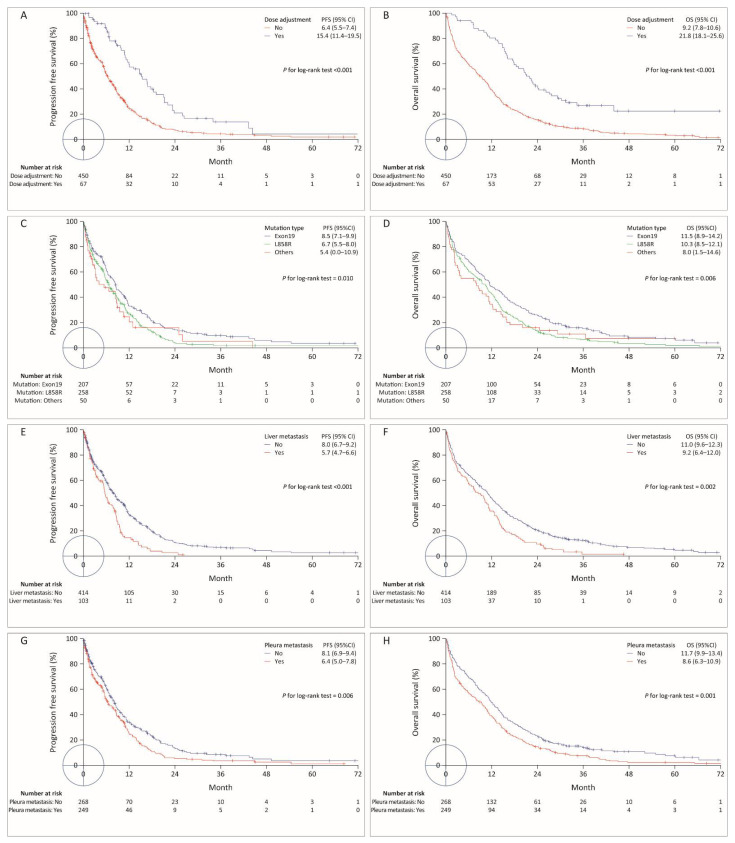
Kaplan–Meier survival curves indicating PFS (**A**,**C**,**E**,**G**) and OS (**B**,**D**,**F**,**H**) among patients stratified according to dose adjustment (**A**,**B**), mutation status (**C**,**D**), liver metastasis (**E**,**F**), and pleural metastasis (**G**,**H**). PFS, progression-free survival; OS, overall survival.

**Figure 4:**
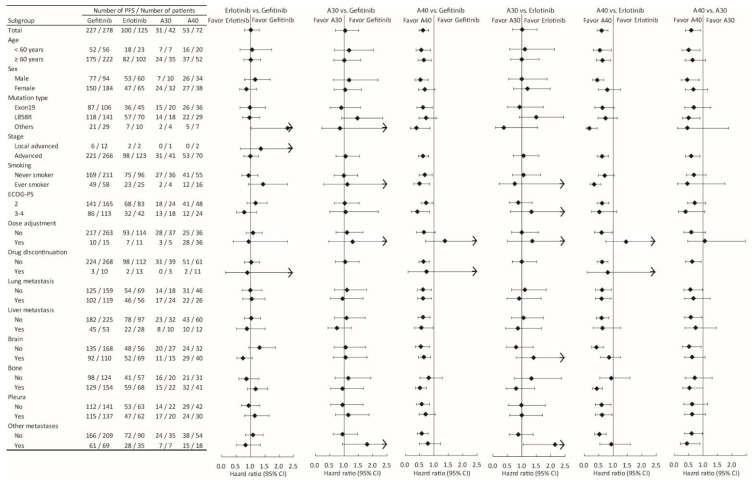
Subgroup analysis comparing progression-free survival among the different epidermal growth factor receptor tyrosine kinase inhibitors treatments, stratified by selected baseline characteristics.

The multivariable model identified potential predictors of PFS, including prescription of 40 mg afatinib (vs. gefitinib, HR: 0.81, 95% CI: 0.56–1.16, *p* = 0.249, Figure 2C), metastatic stage (vs. locally advanced lung cancer, HR: 1.95, 95% CI: 1.12–3.39, *p* = 0.018), dose adjustment (HR: 0.54, 95% CI: 0.40–0.74, *p* < 0.001, Figure 3A), liver metastasis (vs. no liver metastasis, HR: 1.41, 95% CI: 1.11–1.78, Figure 3E), pleural metastasis (vs. no pleural metastasis, HR: 1.25, 95% CI: 1.02–1.53, Figure 3G), L858R mutation (vs. exon 19 deletion, HR: 1.31, 95% CI: 1.05–1.62, *p* = 0.014), and other mutation types (vs. exon 19 deletion, HR: 1.87, 95% CI: 1.30–2.68, *p* = 0.001, Figure 3C). Pairwise comparisons of treatments, stratified according to the selected PFS-associated subgroup variables are shown in Figure 4.

### 3.3. Prognostic Factors for OS

Of the 517 patients, 476 (92.1%) died during a median follow-up of 10.5 months (IQR: 2.5–19.7 months, Figure 2B). A univariate analysis identified potential prognostic factors for OS in patients receiving TKIs. Patients receiving 40 mg afatinib (vs. gefitinib, HR: 0.55, 95% CI: 0.41–0.75, *p* ≤ 0.001) or underwent dose adjustment (HR: 0.42, 95% CI: 0.33–0.54, *p* < 0.001) had favorable OS outcomes. Patients with age ≥60 years (vs. <60 years, HR: 1.30, 95% CI: 1.06–1.61, *p* = 0.013), L858R (vs. exon 19 deletion, HR:1.35, 95% CI: 1.11–1.63, *p* = 0.002) or uncommon mutations (vs. exon 19 deletion, HR:1.40, 95% CI: 0.96–2.04, *p* = 0.082), metastatic (vs. local advanced, HR: 1.82, 95% CI: 1.20–2.77, *p* = 0.005), lung metastasis (vs. no lung metastasis, HR:1.28, 95% CI: 1.07–1.54, *p* = 0.008), liver metastasis (vs. no liver metastasis, HR: 1.44, 95% CI: 1.16–1.77, *p* = 0.001), or pleural metastasis (vs. no pleural metastasis, HR:1.37, 95% CI:1.08–1.60, *p* = 0.001) had unfavorable OS outcomes (Table 3).

**Figure 5:**
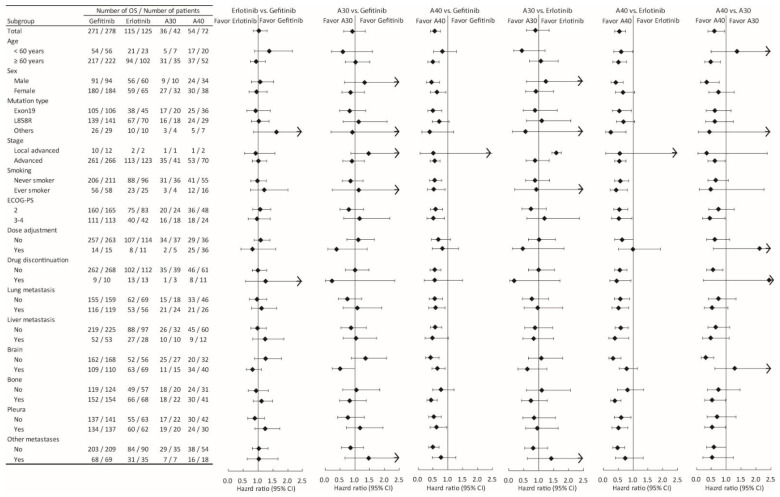
Subgroup analysis comparing overall survival among the different epidermal growth factor receptor tyrosine kinase inhibitors treatments, stratified by selected baseline characteristics.

The multivariable model identified the following potential predictive factors for OS, including the prescription of 40 mg afatinib (HR 0.79, 95% CI 0.56–1.12, not significant, Figure 2D), age ≥ 60 years (HR: 1.42, 95% CI: 1.12–1.80, *p* = 0.004), dose adjustment (HR: 0.48, 95% CI: 0.36–0.64, *p* < 0.001, Figure 3B), lung metastasis (vs. no lung metastasis, HR: 1.22, 95% CI: 1.01–1.47, *p* = 0.039), liver metastasis (vs. no liver metastasis, HR: 1.38, 95% CI: 1.13–1.68, *p* = 0.002, Figure 3F), pleural metastasis (vs. no pleural metastasis, HR: 1.22, 95% CI: 1.01–1.47, *p* = 0.039, Figure 3H), L858R mutation (vs. exon 19 deletion, HR: 1.28, 95% CI: 1.05–1.56, *p* = 0.014), and other mutation types (vs. exon 19 deletion, HR: 1.79, 95% CI: 1.32–2.42, *p* < 0.0001, Figure 3D). The pairwise comparisons of treatments stratified by selected OS-associated subgroup variables are shown in Figure 5.

### 3.4. Subgroup Analyses for PFS and OS

To further identify possible predictive factors for the use of various EGFR-TKIs, subgroup analyses were performed (Figure 4 and Figure 5). The superiority of 40 mg afatinib was evident in all specified patient subgroups, except for those stratified by dose adjustment and drug discontinuation.

### 3.5. Adverse Events

**Figure 6:**
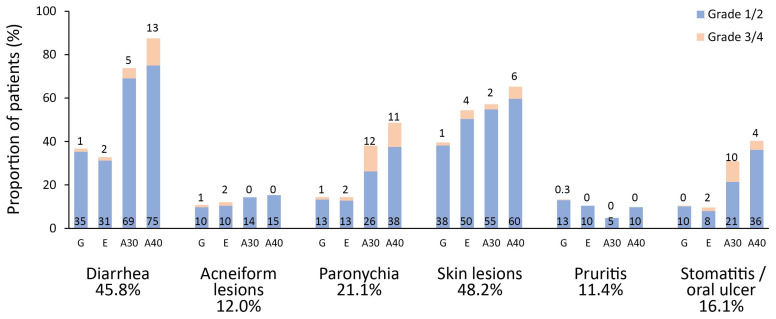
The distribution of adverse events associated with the different epidermal growth factor receptor tyrosine kinase inhibitors treatments. Only adverse events >10% at all grades are shown.

The distribution of AEs (≥10%) according to treatment groups is displayed in Figure 6. The most frequently reported AE was diarrhea (45.8%), then skin lesions (48.2%), paronychia (21.1%), and stomatitis/oral ulcer (16.1%). Most AEs were considered mild (Grade 1 or 2) and manageable. In terms of severe AEs (Grade ≥ 3), paronychia (3.5%) was the most frequently reported event, followed by diarrhea (3.3%), skin rashes (2.7%), and stomatitis/oral ulcer (1.9%). Overall, AE grades, including severe AEs, were more likely to be associated with either 30 or 40 mg afatinib (particularly 40 mg afatinib) compared with gefitinib treatment (Figure 6 and Tables S2 and S3).

### 3.6. Subsequent Treatment after EGFR-TKIs

Overall, 178 (34.4%) patients received subsequent treatments after the failure of first-line EGFR-TKIs, including chemotherapy (*n* = 136, 26.3%), TKIs other than osimertinib (*n* = 95, 18.4%), osimertinib (*n* = 19, 3.7%), bevacizumab (*n* = 13, 2.5%), and immune checkpoint inhibitors (*n* = 5, 1.0%). The low proportion of subsequent treatment in the current study reflects the nature of patients with poor PS. Those patients receiving 40 mg afatinib were more likely to receive subsequent treatment with osimertinib (11.1%), bevacizumab (6.9%), and immune checkpoint inhibitors (2.8%) than those patients treated with gefitinib (Table 4).

## 4. Discussion

This study represents the first large cohort study to evaluate the efficacy and tolerability of EGFR-TKIs in EGFR-mutated lung adenocarcinoma patients with PS ≥ 2, as such patients are typically not included in clinical trials. The data demonstrated that EGFR-TKIs are effective and well-tolerated in lung adenocarcinoma patients with poor PS. Although 22.4% of patients had no response, indicating the severe nature of the disease in such patients, the overall response rate was 56.3%. The median PFS and OS values were 11.4 months (95% CI: 10.0–12.8 months) and 15.3 months (95%CI: 13.7–16.9 months), respectively. Patients treated with 40 mg afatinib had better survival rates than patients treated with other TKIs, although this superiority was not significant in the multivariate analysis. Importantly, mutation status, dose adjustment, and the presence of liver and pleural metastases were independent prognostic factors for both PFS and OS (Table 2 and Table 3). As expected, patients treated with afatinib, particularly at the 40 mg dose, experienced more all-Grade and Grade 3/4 AEs, and only 178 (34.4%) patients received subsequent treatments.

Patients being treated with 40 mg afatinib were significantly younger than other patients, suggesting that physicians prefer the use of TKIs for EGFR-mutated lung adenocarcinoma patients. Although afatinib demonstrated better efficacy than gefitinib in the LUX-Lung 7 Phase 2 study [16], more frequent toxicity, including diarrhea, skin rashes, and stomatitis, was reported in patients treated with afatinib, limiting the clinical use of afatinib in patients with poor PS. Therefore, some retrospective studies examining limited numbers of patients with poor PS described the use of 30 mg afatinib daily as the starting dose, as an alternative to the standard 40 mg dose, and the lower dose was associated with reduced toxicity and better tolerance without compromising the efficacy [24,25]. In addition to age, more patients with brain metastasis were treated with 40 mg afatinib, implying the potential importance of dose intensity for controlling brain metastases. Another study showed that the superior efficacy of 40 mg afatinib was limited among patients with brain metastasis [26]. However, the superior efficacy of 40 mg afatinib was found in patients irrespective of brain metastasis in the PFS analysis of the current study, indicating that brain metastasis is not a predictive factor for the use of 40 mg afatinib (Figure 4).

Overall, the 40 mg afatinib treatment was associated with the best PFS and OS in the current cohort. In the subgroup analyses (Figure 4 and Figure 5), this superiority was found for all subgroups, except those stratified by dose adjustment and drug discontinuation. Interestingly, no differences in either PFS or OS were found among patients who experienced dose adjustment in response to AEs during EGFR-TKI treatment (Figure 4 and Figure 5). In addition, the need for dose adjustment was the most significant prognostic factor for both PFS and OS (Table 2, Figure 3A,E). These findings implied that the patients who experience AEs and require dose adjustments might respond better to all EGFR-TKIs. This finding is comparable to the literature demonstrating that patients undergoing dacomitinib with dose reduction had better PFS and OS than those without dose reduction. This could be explained in that the patients requiring dose reduction had a higher initial plasma concentration than those without dose reduction [27]. In our previous study of afatinib in patients with poor PS, dose adjustment was associated with DCR but not significantly associated with PFS and OS possibly due to limited cases (62 cases) [28].

In contrast, among patients who experienced drug discontinuation, a similar trend of no differences among EGFR-TKIs was found for PFS but not for OS, which might be influenced by the sequential treatment with other EGFR-TKIs [29,30]. These findings should be carefully interpreted, as a limited proportion of patients experienced either dose adjustment or drug discontinuation in the current cohort, except in the 40 mg afatinib group due to the higher level of AEs compared with other EGFR-TKIs [24]. The low proportion of dose adjustments and drug discontinuations likely reflects the fact that EGFR-TKIs are generally well-tolerated, resulting in few patients requiring drug discontinuation. Although 50% of patients who received 40 mg afatinib treatment required dose adjustments due to AEs, most patients were able to tolerate afatinib, as only 15.3% of patients required drug discontinuation. Neither dose adjustment nor drug discontinuation appeared to compromise the efficacy of EGFR-TKIs and may represent a biomarker to predict better survival of patients.

In addition to dose adjustment, the mutation status and the presence of liver and pleural metastases were independently unfavorable prognostic factors (Figure 3 and Table 2 and Table 3). Lung adenocarcinoma patients who harbor the exon 19 deletion are known to present with better survival than patients with the exon 21 L858R mutation [31,32]. Patients with liver and pleural metastases had worse survival outcomes, which agrees with the outcomes of previous studies [33,34,35].

The major limitation of this study was the retrospective nature of this study; however, from the literature, this study is the biggest cohort study to enroll patients with PS ≥ 2 who were treated with EGFR-TKIs. The poor PS may result from lung cancer itself or underlying comorbidities, which were difficult to determine, which is a limitation of this study. A total of 22.4% patients did not have tumor evaluation because of rapid progression of tumor or patients’ fragility after the initiation of EGFR-TKIs. AEs may not have been recorded accurately; however, the overall frequency of AEs was comparable with those reported by previous clinical trials.

## 5. Conclusions

In conclusion, this study showed the real-world experience of EGFR-TKI use as a first-line treatment in EGFR-mutated lung adenocarcinoma patients with poor PS. This study demonstrated that EGFR-TKIs are well-tolerated in patients with poor PS and have comparable anti-tumor activity. Dose adjustment was an independent prognostic factor for PFS and OS. Unfortunately, only one-third of patients received subsequent treatment, suggesting that patients experienced only a one-line option for lung cancer treatment. Current research provided novel evidence to support the clinical use of EGFR-TKIs for patients with poor PS. More studies are warranted to validate our findings.

## Figures and Tables

**Table 1:** Baseline characteristics.

Variable	All Patients (*n* = 517)	Gefitinib (*n* = 278)	Erlotinib (*n* = 125)	Afatinib 30 mg (*n* = 42)	Afatinib 40 mg (*n* = 72)	*p* Value
Age, years	73.1 (61.9, 80.3)	75.1 (62.0, 81.2)	72.0 (63.1, 80.5)	73.4 (64.1, 79.3)	65.2 (58.9, 76.0) ^a,b^	0.003
Age group						0.380
<60 years	106 (20.5)	56 (20.1)	23 (18.4)	7 (16.7)	20 (27.8)	
≥60 years	411 (79.5)	222 (79.9)	102 (81.6)	35 (83.3)	52 (72.2)	
Male	198 (38.3)	94 (33.8)	60 (48.0) ^a^	10 (23.8) ^b^	34 (47.2)	0.004
Mutation type						0.383
Exon19	207 (40.2)	106 (38.4)	45 (36.0)	20 (47.6)	36 (50.0)	
L858R	258 (50.1)	141 (51.1)	70 (56.0)	18 (42.9)	29 (40.3)	
Others	50 (9.7)	29 (10.5)	10 (8.0)	4 (9.5)	7 (9.7)	
Stage						0.530
Locall advanced (IIIB)	17 (3.3)	12 (4.3)	2 (1.6)	1 (2.4)	2 (2.8)	
Metastatic (IV)	500 (96.7)	266 (95.7)	123 (98.4)	41 (97.6)	70 (97.2)	
Smoking group						0.659
Never smoker	398 (77.0)	211 (75.9)	96 (76.8)	36 (85.7)	55 (76.4)	
Ever smoker	103 (19.9)	58 (20.9)	25 (20.0)	4 (9.5)	16 (22.2)	
Unknown	16 (3.1)	9 (3.2)	4 (3.2)	2 (4.8)	1 (1.4)	
ECOG-PS						0.402
2	320 (61.9)	165 (59.4)	83 (66.4)	24 (57.1)	48 (66.7)	
3–4	197 (38.1)	113 (40.6)	42 (33.6)	18 (42.9)	24 (33.3)	
Dose adjustment	67 (13.0)	15 (5.4)	11 (8.8)	5 (11.9)	36 (50.0) ^a^	<0.001
Drug discontinuation	37 (7.2)	10 (3.6)	13 (10.4) ^a^	3 (7.1)	11 (15.3) ^a^	0.002
Metastatic sites						
Lung	225 (43.5)	119 (42.8)	56 (44.8)	24 (57.1)	26 (36.1)	0.178
Liver	103 (19.9)	53 (19.1)	28 (22.4)	10 (23.8)	12 (16.7)	0.686
Brain	234 (45.3)	110 (39.6)	69 (55.2) ^a^	15 (35.7)	40 (55.6)	0.004
Bone	285 (55.1)	154 (55.4)	68 (54.4)	22 (52.4)	41 (56.9)	0.968
Pleura	249 (48.2)	137 (49.3)	62 (49.6)	20 (47.6)	30 (41.7)	0.690
Other sites	129 (25.0)	69 (24.8)	35 (28.0)	7 (16.7)	18 (25.0)	0.539
Tumor response						0.287
Partial response	291 (56.3)	156 (56.1)	63 (50.4)	26 (61.9)	46 (63.9)	
Stable disease	59 (11.4)	30 (10.8)	16 (12.8)	3 (7.1)	10 (13.9)	
Progressive disease	51 (9.9)	32 (11.5)	12 (9.6)	1 (2.4)	6 (8.3)	
Not available	116 (22.4)	60 (21.6)	34 (27.2)	12 (28.6)	10 (13.9)	

Abbreviations: ECOG-PS: Eastern Cooperative Oncology Group Performance Status; “^a^” indicates *p* < 0.05 vs. Gefitinib; “^b^” indicates *p* < 0.05 vs. Erlotinib. Data were presented as frequency (percentage) or median (25th percentile, 75th percentile).

**Table 2:** Univariate and multivariable analysis for the associated factors with progression-free survival.

	Univariate Analysis		Multivariable Analysis *	
Predictor	HR (95% CI)	*p*	HR (95% CI)	*p*
TKI				
Gefitinib	Reference		Reference	
Erlotinib	1.02 (0.80–1.31)	0.851	1.05 (0.82–1.35)	0.693
Afatinib 30 mg	1.04 (0.71–1.51)	0.852	1.11 (0.77–1.60)	0.588
Afatinib 40 mg	0.62 (0.47–0.81)	0.001	0.81 (0.56–1.16)	0.249
Age				
<60 years	Reference			
≥60 years	1.01 (0.82–1.24)	0.966		
Sex				
Male	Reference			
Female	0.88 (0.72–1.07)	0.199		
Mutation type				
Exon19	Reference		Reference	
L858R	1.34 (1.09–1.64)	0.006	1.31 (1.05–1.62)	0.014
Others	1.47 (0.99–2.17)	0.054	1.87 (1.30–2.68)	0.001
Stage				
Locall advanced (IIIB)	Reference		Reference	
Metastatic (IV)	2.76 (1.48–5.15)	0.002	1.95 (1.12–3.39)	0.018
Smoking				
Never smoker	Reference			
Ever smoker	1.04 (0.82–1.32)	0.742		
Unknown	1.20 (0.83–1.73)	0.339		
ECOG-PS				
2	Reference			
3–4	1.01 (0.82–1.25)	0.916		
Dose adjustment	0.46 (0.36–0.59)	<0.001	0.54 (0.40–0.74)	<0.001
Drug discontinuation	0.38 (0.16–0.87)	0.021	0.47 (0.20–1.07)	0.070
Metastatic sites				
Lung	1.29 (1.06–1.56)	0.011	1.19 (0.98–1.46)	0.080
Liver	1.59 (1.27–1.99)	<0.001	1.41 (1.11–1.78)	0.004
Brain	1.19 (0.98–1.44)	0.081	1.18 (0.97–1.45)	0.106
Bone	1.15 (0.95–1.40)	0.154		
Pleura	1.32 (1.08–1.60)	0.006	1.25 (1.02–1.53)	0.033
Other sites	1.26 (1.01–1.57)	0.039	1.14 (0.91–1.43)	0.255

Abbreviations: CI: confidence interval; ECOG-PS: Eastern Cooperative Oncology Group Performance Status; HR: hazard ratio; TKI: tyrosine kinase inhibitor. * Those variables with significance values less than 0.15 in the univariate Cox analysis were further introduced into a multivariable Cox model.

**Table 3:** Univariate and multivariable analysis for the associated factors with overall survival.

	Univariate Analysis		Multivariable Analysis *	
Predictor	HR (95% CI)	*p*	HR (95% CI)	*p*
TKI				
Gefitinib	Reference		Reference	
Erlotinib	1.04 (0.83–1.30)	0.767	1.07 (0.85–1.34)	0.561
Afatinib 30mg	0.92 (0.63–1.35)	0.677	1.00 (0.70–1.42)	0.993
Afatinib 40mg	0.55 (0.41–0.75)	<0.001	0.79 (0.55–1.12)	0.186
Age				
<60 years	Reference		Reference	
≥60 years	1.30 (1.06–1.61)	0.013	1.42 (1.12–1.80)	0.004
Sex				
Male	Reference			
Female	0.88 (0.72–1.06)	0.175		
Mutation type				
Exon19	Reference		Reference	
L858R	1.35 (1.11–1.63)	0.002	1.28 (1.05–1.56)	0.014
Others	1.40 (0.96–2.04)	0.082	1.79 (1.32–2.42)	<0.0001
Stage				
Locall advanced (IIIB)	Reference		Reference	
Metastatic (IV)	1.82 (1.20–2.77)	0.005	1.50 (0.96–2.34)	0.076
Smoking				
Never smoker	Reference			
Ever smoker	1.00 (0.80–1.25)	0.986		
Unknown	1.28 (0.91–1.81)	0.154		
ECOG-PS				
2	Reference		Reference	
3–4	1.19 (0.99–1.44)	0.066	1.18 (0.97–1.43)	0.091
Dose adjustment	0.42 (0.33–0.54)	<0.001	0.48 (0.36–0.64)	<0.001
Drug discontinuation	0.86 (0.60–1.24)	0.422		
Metastatic sites				
Lung	1.28 (1.07–1.54)	0.008	1.22 (1.01–1.47)	0.039
Liver	1.44 (1.16–1.77)	0.001	1.38 (1.13–1.68)	0.002
Brain	1.11 (0.93–1.33)	0.242		
Bone	1.12 (0.94–1.35)	0.219		
Pleura	1.37 (1.14–1.64)	0.001	1.22 (1.01–1.47)	0.039
Other sites	1.11 (0.90–1.37)	0.328		

Abbreviations: CI: confidence interval; ECOG-PS: Eastern Cooperative Oncology Group Performance Status; HR: hazard ratio; TKI: tyrosine kinase inhibitor. * Those variables with significance values less than 0.15 in the univariate Cox analysis were further introduced into a multivariable Cox model

**Table 4:** Subsequent treatment.

Variable	All Patients (*n* = 517)	Gefitinib (*n* = 278)	Erlotinib (*n* = 125)	Afatinib 30 mg (*n* = 42)	Afatinib 40 mg (*n* = 72)	*p* Value
Any subsequent treatment	178 (34.4)	93 (33.5)	37 (29.6)	14 (33.3)	34 (47.2)	0.084
Chemotherapy	136 (26.3)	76 (27.3)	29 (23.2)	10 (23.8)	21 (29.2)	0.764
Tyrosine kinase inhibitor	95 (18.4)	52 (18.7)	20 (16.0)	8 (19.0)	15 (20.8)	0.839
Osimertinib	19 (3.7)	4 (1.4)	7 (5.6)	0 (0.0)	8 (11.1) ^a^	0.001
Bevacizumab	13 (2.5)	0 (0.0)	6 (4.8) ^a^	2 (4.8) ^a^	5 (6.9) ^a^	<0.001
Immunotherapy	5 (1.0)	0 (0.0)	2 (1.6)	1 (2.4)	2 (2.8) ^a^	0.026
Any subsequent treatment	178 (34.4)	93 (33.5)	37 (29.6)	14 (33.3)	34 (47.2)	0.084

Abbreviations: “^a^” indicates *p* < 0.05 vs. Gefitinib.

## Data Availability

The data presented in this study are available on request from the corresponding author.

## Supplementary Materials

The following supporting information can be downloaded at: https://www.mdpi.com/article/10.3390/cancers14030674/s1, Table S1. The distribution of detailed metastatic sites; Table S2. Adverse events for any grades; Table S3. Adverse events for grades 3–4.
