# Supplementary material for: Comparison of Different Tyrosine Kinase Inhibitors for Treatment of Poor Performance Status Patients with EGFR-Mutated Lung Adenocarcinoma
Source: Cancers (Basel). 2022 Jan 28;14(3):674. doi: 10.3390/cancers14030674 (PMC8833436; doi:10.3390/cancers14030674)
Supplement: Supplementary file 1 [file cancers-14-00674-s001.zip › cancers-1568102-supplementary.pdf]

cancers MDPI

Supplementary Materials

Table S1. The distribution of detailed metastatic sites.

Variable	All Patients (n = 517)	Gefitinib (n = 278)	Erlotinib (n = 125)	Afatinib 30 mg (n = 42)	Afatinib 40 mg (n = 72)
Lung	225 (43.5)	119 (42.8)	56 (44.8)	24 (57.1)	26 (36.1)
Liver	103 (19.9)	53 (19.1)	28 (22.4)	10 (23.8)	12 (16.7)
Brain	234 (45.3)	110 (39.6)	69 (55.2)	15 (35.7)	40 (55.6)
Bone	285 (55.1)	154 (55.4)	68 (54.4)	22 (52.4)	41 (56.9)
Pleura	249 (48.2)	137 (49.3)	62 (49.6)	20 (47.6)	30 (41.7)
Adrenal	50 (9.7)	23 (8.3)	17 (13.6)	3 (7.1)	7 (9.7)
Distal lymph	61 (11.8)	33 (11.9)	14 (11.2)	5 (11.9)	9 (12.5)
Peritoneum	3 (0.6)	0 (0.0)	1 (0.8)	0 (0.0)	2 (2.8)
Pericardium	25 (4.8)	12 (4.3)	10 (8.0)	1 (2.4)	2 (2.8)
Spleen	9 (1.7)	8 (2.9)	0 (0.0)	0 (0.0)	1 (1.4)

Table S2. Adverse events for any grades.

Variable	All Patients (n = 517)	Gefitinib (n = 278)	Erlotinib (n = 125)	Afatinib 30 mg (n = 42)	Afatinib 40 mg (n = 72)	p Value
Any adverse event	424 (82.0)	221 (79.5)	94 (75.2)	39 (92.9)	70 (97.2) [a,b]	<0.001
Diarrhea lesions	237 (45.8)	102 (36.7)	41 (32.8)	31 (73.8) [a,b]	63 (87.5) [a,b]	<0.001
Acneiform	62 (12.0)	30 (10.8)	15 (12.0)	6 (14.3)	11 (15.3)	0.662
Paronychia	109 (21.1)	40 (14.4)	18 (14.4)	16 (38.1) [a,b]	35 (48.6) [a,b]	<0.001
Skin lesions	249 (48.2)	110 (39.6)	68 (54.4) [a]	24 (57.1)	47 (65.3) [a]	<0.001
Pruritis	59 (11.4)	37 (13.3)	13 (10.4)	2 (4.8)	7 (9.7)	0.418
Nausea and vomiting	51 (9.9)	29 (10.4)	11 (8.8)	4 (9.5)	7 (9.7)	0.980
Constipation	51 (9.9)	40 (14.4)	6 (4.8) a	1 (2.4)	4 (5.6)	0.003
Dry skin (Xerosis)	38 (7.4)	21 (7.6)	10 (8.0)	3 (7.1)	4 (5.6)	0.945
Stomatitis / oral ulcer	83 (16.1)	29 (10.4)	12 (9.6)	13 (31.0) [a,b]	29 (40.3) [a,b]	<0.001
Hair loss	4 (0.8)	2 (0.7)	2 (1.6)	0 (0.0)	0 (0.0)	0.732
Hand-foot skin reaction	10 (1.9)	5 (1.8)	2 (1.6)	1 (2.4)	2 (2.8)	0.808
Eyes	10 (1.9)	7 (2.5)	0 (0.0)	0 (0.0)	3 (4.2)	0.111
Interstitial lung disease	14 (2.7)	9 (3.2)	3 (2.4)	0 (0.0)	2 (2.8)	0.853
Abnormal liver function test	7 (1.4)	3 (1.1)	2 (1.6)	0 (0.0)	2 (2.8)	0.508
Edema	43 (8.3)	30 (10.8)	7 (5.6)	4 (9.5)	2 (2.8)	0.087
Onchynosis	1 (0.2)	0 (0.0)	0 (0.0)	0 (0.0)	1 (1.4)	0.221
Fatigue	6 (1.2)	2 (0.7)	2 (1.6)	2 (4.8)	0 (0.0)	0.115

Abbreviations: "[a]" indicates $p < 0.05$ vs. Gefitinib; "[b]" indicates $p < 0.05$ vs. Erlotinib.

Table S3. Adverse events for grades 3–4.

Variable	All Patients (*n* = 517)	Gefitinib (*n* = 278)	Erlotinib (*n* = 125)	Afatinib 30 mg (*n* =4 2)	Afatinib 40 mg (*n* = 72)	*p* Value
Any adverse event	55 (10.6)	14 (5.0)	13 (10.4)[a]	10 (23.8)[a,b]	18 (25.0)[a,b]	<0.001
Diarrhea	17 (3.3)	4 (1.4)	2 (1.6)	2 (4.8)	9 (12.5)[a,b,c]	<0.001
Acneiform lesions	5 (1.0)	3 (1.1)	2 (1.6)	0 (0.0)	0 (0.0)	0.794
Paronychia	18 (3.5)	3 (1.1)	2 (1.6)	5 (11.9)[a,b]	8 (11.1)[a,b]	<0.001
Skin lesions	14 (2.7)	4 (1.4)	5 (4.0)	1 (2.4)	4 (5.6)	0.113
Pruritis	1 (0.2)	1 (0.4)	0 (0.0)	0 (0.0)	0 (0.0)	1.000
Nausea and vomiting	0 (0.0)	0 (0.0)	0 (0.0)	0 (0.0)	0 (0.0)	NA
Constipation	0 (0.0)	0 (0.0)	0 (0.0)	0 (0.0)	0 (0.0)	NA
Dry skin (Xerosis)	0 (0.0)	0 (0.0)	0 (0.0)	0 (0.0)	0 (0.0)	NA
Stomatitis / oral ulcer	10 (1.9)	1 (0.4)	2 (1.6)	4 (9.5)[a]	3 (4.2)[a]	<0.001
Hair loss	0 (0.0)	0 (0.0)	0 (0.0)	0 (0.0)	0 (0.0)	NA
Hand-foot skin reaction	1 (0.2)	0 (0.0)	0 (0.0)	0 (0.0)	1 (1.4)	0.221
Eyes	0 (0.0)	0 (0.0)	0 (0.0)	0 (0.0)	0 (0.0)	NA
Interstitial lung disease	1 (0.2)	1 (0.4)	0 (0.0)	0 (0.0)	0 (0.0)	1.000
Abnormal liver function test	2 (0.4)	1 (0.4)	1 (0.8)	0 (0.0)	0 (0.0)	0.711
Edema	1 (0.2)	0 (0.0)	1 (0.8)	0 (0.0)	0 (0.0)	0.462
Onchynosis	0 (0.0)	0 (0.0)	0 (0.0)	0 (0.0)	0 (0.0)	NA
Fatigue	0 (0.0)	0 (0.0)	0 (0.0)	0 (0.0)	0 (0.0)	NA

Abbreviations: "a" indicates $p < 0.05$ vs. Gefitinib; "b" indicates $p < 0.05$ vs. Erlotinib; "c" indicates $p < 0.05$ vs. Afatinib 30 mg.
